# Thioredoxin-Interacting Protein Mediates NLRP3 Inflammasome Activation Involved in the Susceptibility to Ischemic Acute Kidney Injury in Diabetes

**DOI:** 10.1155/2016/2386068

**Published:** 2016-10-27

**Authors:** Ye Da Xiao, Ya Yi Huang, Hua Xin Wang, Yang Wu, Yan Leng, Min Liu, Qian Sun, Zhong-Yuan Xia

**Affiliations:** Department of Anesthesiology, Renmin Hospital of Wuhan University, 99 Zi Yang Road, Wuhan, Hubei 430060, China

## Abstract

Kidney in diabetic state is more sensitive to ischemic acute kidney injury (AKI). However, the underlying mechanisms remain unclear. Herein, we examined the impact of diabetes mellitus on thioredoxin-interacting protein (TXNIP) expression and whether mediated NLRP3 activation was associated with renal ischemia/reperfusion- (I/R-) induced AKI. In an* in vivo* model, streptozotocin-induced diabetic rats showed higher susceptibility to I/R injury with increased TXNIP expression, which was significantly attenuated by resveratrol (RES) treatment (10 mg/kg intraperitoneal daily injection for 7 consecutive days prior to I/R induction). RES treatment significantly inhibited TXNIP binding to NLRP3 in diabetic rats subjected to renal I/R injury. Furthermore, RES treatment significantly reduced cleaved caspase-1 expression and production of IL-1*β* and IL-18. In an* in vitro* study using cultured human kidney proximal tubular cell (HK-2 cells) in high glucose condition (HG, 30 mM) subjected to hypoxia/reoxygenation (H/R), HG combined H/R (HH/R) stimulated TXNIP expression which was accompanied by increased NLRP3 expression, ROS generation, caspase-1 activity and IL-1*β* levels, and aggravated HK-2 cells apoptosis. All these changes were significantly attenuated by TXNIP RNAi and RES treatment. In conclusion, our results demonstrate that TXNIP-mediated NLRP3 activation through oxidative stress is a key signaling mechanism in the susceptibility to AKI in diabetic models.

## 1. Introduction

It is estimated that 1-2% of new hospital admissions and 2–7% of cases acquired during hospital stays are due to acute kidney injury (AKI) [1]. The major risk factors for AKI include diabetes mellitus (DM), hypertension, and congestive heart failure [2]. In developed countries, DM is the primary cause of chronic kidney disease (CKD) and diabetic nephropathy (DN) is an important complication of diabetic patients [3]. Furthermore, studies have shown that hyperglycemia induces oxidative stress in kidney cells [4, 5]. The major causes of AKI include ischemia, hypoxia, or nephrotoxicity [6]. Diabetic patients are also at a risk of requiring hospitalization and undergoing AKI [7], but the underlying mechanisms remain unknown. Studies in rat models of type-1 diabetes indicated that diabetic rats had increased susceptibility to AKI compared to nondiabetic rats [8, 9]. Another research study suggested that inflammation was involved in the mechanism [10], while Gao et al. further demonstrated that TNF-*α* mediated the increased susceptibility to ischemic AKI in diabetes [11].

The Nod-like receptor protein 3 (NLRP3) inflammasome, a key mediator of the innate immune system in response to a host of initiating factors, is activated in response to various diseases [12–14]. The NLRP3 inflammasome is composed of apoptosis-associated speck-like adaptor protein containing a CARD (caspase recruitment domain) (ASC), oligomers of the receptor (NLRP3), and pro-caspase-1. Upon activation, NLRP3 is ligated with ASC, which in turn combines pro-caspase-1, causing its transformation to cleaved caspase-1 that regulates the maturation of proinflammatory cytokines IL-1*β* and IL-18 [15, 16]. Recent studies have demonstrated that NLRP3 contributes to renal ischemia/reperfusion (I/R) injury* via* a direct effect on renal tubular epithelium [17, 18]. However the signaling pathways that lead to the activation of NLRP3 inflammasome due to kidney I/R injury have not been fully elucidated. Many activators of the NLRP3 inflammasome have been identified, including K^+^ channels, lysosomal membrane, and ROS [19]. A previous study determined that ROS activity played an important role in AKI with models of I/R [6]. Meanwhile, the production of ROS and the generation of oxidative stress are important elements for the pathophysiology of DM [20]. Hyperglycemia induced generation of ROS in renal tubular epithelial cells in* in vitro* studies [21, 22]. However, the mechanisms by which ROS activates the inflammasome are unclear. Recently, the significant work by Zhou et al. revealed that thioredoxin-interacting protein (TXNIP) is an upstream partner to NLRP3 and that the association between these two proteins was necessary for downstream inflammasome activation [23].

TXNIP, the endogenous inhibitor and regulator of TRX, is a major cellular antioxidant and antiapoptotic protein [24]. Overexpression of TXNIP inhibits the activity of TRX and thus can modulate the cellular redox state and stimulate oxidative stress [25, 26]. In addition, experimental evidence has indicated that hyperglycemia can induce TXNIP expression in renal tubular epithelial cells [21, 22], and diabetes can potentially enhance TXNIP expression and reduce TRX activity [27]. However, there is no direct evidence to support a causative role of TXNIP in hyperglycemia or high blood glucose which exasperates renal I/R injury.

Resveratrol (trans-3,4,5-trihydroxystilbene, RES), a natural polyphenolic mixture concentrated in grape skin and red wine [28], is reported to have beneficial effects on renal diseases. Resveratrol is reported to be a strong scavenger of ROS [29]. RES treatment can ameliorate hyperglycemia-mediated renal dysfunction or DN [30]. Several studies have demonstrated that RES exerts protective effects against I/R injury in the kidneys [31], as well as the liver and brain ischemia injury by reducing oxidative stress and downregulating TXNIP expression [32, 33].

The present study was designed to investigate the role of TXNIP during ischemia AKI in diabetic models and examine a key role of TXNIP in bridging redox signals with activation of NLRP3 inflammasome in AKI injury.

## 2. Materials and Methods

### 2.1. Antibodies and Reagents

The following antibodies were used in this study: TXNIP (ab86983, Abcam, UK), NLRP3 (NBP-12446, Novus Biologicals, USA), and caspase-1 and cleaved caspase-1 (Santa-514, Santa Cruz, CA). VDUP-1 (TXNIP) siRNA Plasmid (sc-44943) and control siRNA Plasmid were from Santa Cruz Biotechnology.

### 2.2. Cell Culture and Transfection

HK-2 cells (ATCC, American Type Culture Collection, Manassas, VA) were cultured in MEM medium (#31985, Gibco, Grand Island, USA) supplemented with 10% fetal bovine serum (FBS, #10099-141, Gibco, Grand Island, USA) and 1% Penicillin-Streptomycin solution in 95% air and 5% CO_2_ atmosphere. For siRNA transfections of HK-2 cells (VDUP-1 siRNA or scrambled siRNA as a nonspecific control), cells were seeded (2 × 10^5^ per well) in 6-well plates and transfected using with Lipofectamine 2000 reagent (#11668-019, Invitrogen, USA) according to the manufacturer's instructions. The cells were used for further experiments 48 hours after transfection. After this time period, the cells were randomly divided into eight groups: NG group (stimulated with NG (5.6 mM)); HG group (30 mM); NG + mannitol (24.4 mM) group (M) as an osmotic control; NH/R (hypoxia 4 hours and reoxygenation 2 hours) group; HH/R group; HH/R treated with scrambled siRNA group; HH/R treated with TXNIP siRNA group; and HH/R treated with RES (50 *µ*M) during the 72 hours of high glucose (HG) incubation [34].

### 2.3. Cell* In Vitro* Simulated Ischemia/Reperfusion Model (Hypoxia and Reoxygenation, H/R)

For hypoxic treatment, after 72 h HG stimulation in the absence or presence of RES (50 *µ*M) [22, 34], HK-2 cells were incubated in glucose-free Krebs-Ringer bicarbonate buffer for 4 hours in a hypoxic chamber equilibrated with 5% CO_2_, 1% O_2_, and 94% N_2_. After hypoxic incubation, the cells were returned to full culture medium for 2-hour reoxygenation. Control cells were incubated in normal cell culture incubator with 21% oxygen [35].

### 2.4. Rat Models of Diabetes

Male Sprague-Dawley rats weighing 250–300 g were used (purchased from Beijing HFK Bioscience Co. Ltd., Beijing, China). All procedures involved in animals were approved by the Ethics Committee of Renmin Hospital of Wuhan University. For streptozotocin- (STZ-) induced diabetes, rats were injected with 65 mg/kg body-weight STZ (Sigma-Aldrich, St. Louis, MO, USA). Two weeks after the STZ injection, the animals were considered to have type-1 diabetes if the plasma glucose level was >300 mg/dL and other diabetic features such as polyuria, polydipsia, and hyperphagia were observed [36]. STZ-induced rats were maintained for another 2 weeks before renal I/R. To examine the effect of RES, 10 mg/kg RES (Sigma-Aldrich, St. Louis, MO, USA) was intraperitoneally injected daily for 7 consecutive days before renal I/R [37]. The diabetic and normal rats were randomly divided into six groups of 4–6 rats each: ND sham group (NS); ND I/R group (NI/R); DM sham group (DS); DM + RES sham group (DS-RES); DM I/R group (DI/R); and DM + RES I/R group (DI/R-RES). All animals were maintained in the animal center of Wuhan University within an environment-controlled room (ambient temperature of 25 ± 1°C and a light/dark period of 12 h) with free access to normal chow and water. All the animal experiments were double-blind. The data statistics were unblinded.

### 2.5. Renal Ischemia-Reperfusion

Renal I/R was induced in rats as previously described [38]. Briefly, rats were anesthetized with 60 mg/kg (intraperitoneally) pentobarbital sodium and kept on homeothermic pad to maintain body temperature at 37°C. Kidneys were exposed by abdominal midline incisions, and the renal pedicles were clamped for 25 min to induce ischemia. After ischemia, the clamps were released for 48 h reperfusion. Sham control animals were subjected to identical operation without renal pedicle clamping. Surgical wounds were sutured and rats were given 1 mL of warm saline intraperitoneally and kept in a warm incubator until they regained consciousness. At 48 h after reperfusion, animals were sacrificed and plasma and tissue samples were collected and stored at −80°C until analysis.

### 2.6. Renal Function, Histology, and Apoptosis

BUN and serum creatinine were determined using commercial kits (Jiancheng Biotech, Nanjing, China) to indicate renal function. Renal histology was examined by H&E staining. Histopathological changes were evaluated by the percentage of tubular injury as indicated by tubular epithelial swelling, loss of brush border, vacuolar degeneration, necrotic tubules, cast formation, and desquamation. The degree of kidney damage was estimated using five randomly selected fields for each rat assessed using quantitative analysis with the following criteria: 0, no abnormalities; 1, slight abnormalities (<25%); 2, moderate abnormalities (25 to 50%); 3, severe abnormalities (50 to 75%); and 4, more severe abnormalities (>75%). Histological sections were evaluated by two examiners blinded to the source of the samples. Renal apoptosis was examined by TUNEL assay using the* in situ* Apoptosis Detection kit from Roche Applied Science. TUNEL-positive cells were identified through the nucleus, which was stained either tan or brown. Five fields were randomly selected and the apoptosis index was calculated as the ratio of apoptotic-to-total cells.

### 2.7. Measurement of Inflammatory Cytokines and Caspase-1 Activity

IL-1*β* and IL-18 levels in kidney were assessed using a rat ELISA kit (Elabscience Biotechnology Co., Ltd., Wuhan, China) according to the manufacturer's instructions. Caspase-1 activity in HK-2 cells was determined using an enzyme activity assay kit (Beyotime Biotechnology, Shanghai, China), according to the manufacturer's instructions.

### 2.8. Measurement of Oxidative Stress

The intracellular formation of ROS was detected using the fluorescence probe 2′,7′-dichlorodihydrofluorescein diacetate (DCHF-DA, Jiancheng Biotech, Nanjing, China). Cells were incubated with 1 mmol/L DCHF-DA for 30 min at 37°C and washed in PBS 3 times, and the fluorescence intensity was measured using a fluorometer with excitation at 485 nm and emission at 525 nm. The malondialdehyde (MDA) levels, superoxide dismutase (SOD), and superoxide anion radical scavenging capacity were detected using commercially available kits (Jiancheng Biotech, Nanjing, China), according to the manufacturer's instructions. The detection of superoxide anion radical scavenging capacity was* via* a spectrophotometer test. Briefly, kidney samples were harvested into PBS buffer and homogenized under high-speed cryogenic centrifugation and the supernatant was extracted. The extract and corresponding reagent were mixed in a constant temperature water bath at 37°C for 40 minutes, before the color reagent was added and allowed to incubate for 10 min. Distilled water was used to calibrate the spectrophotometer and the absorbance value of each sample at wavelength 550 nm with an optical diameter of 1 cm was obtained.

### 2.9. Immunofluorescence Staining

HK-2 cells were seeded on cover slips and were later subjected to fixation with 4% paraformaldehyde for 15 min and permeabilization in 0.5% Triton X-100 (Beyotime, Shanghai, China: ST795) for 20 min at room temperature. Then the cells were blocked with normal goat serum (Boster, Wuhan, China: AR1009) for 30 min at room temperature and incubated with primary antibody against TXNIP (1 : 50) and NLRP3 (1 : 50) overnight at 4°C. After incubation with secondary antibody (Boster, Wuhan, China: BA1032) for 1 hour at 20–37°C, the cover slips were washed with PBS and stained with DAPI (Beyotime, Shanghai, China: C1002). Under 400 (200)x magnification, images were taken by fluorescence microscope (Olympus, Japan).

### 2.10. Immunohistochemical Staining

The IHC staining for TXNIP expression in renal tissues was performed on formalin-fixed, paraffin-embedded samples; 4 *μ*M sections were deparaffinized in graded xylene-alcohol solutions. Subsequently, the samples were subjected to antigen retrieval and then incubated in 3% H_2_O_2_ 15 min and washed by PBS. The sections were incubated overnight (15 hours) at 4°C with a primary antibody anti-TXNIP (1 : 50) and then incubated with horseradish peroxidase-conjugated anti-IgG secondary antibody for 20–30 min. The reaction was visualized with a solution of diaminobenzidine (DAB) and counterstained with hematoxylin.

### 2.11. Western Blot Analysis

The expressions of TXNIP, NLRP3, caspase-1, and cleaved caspase-1 were examined using Western blot. Protein content was determined with BCA protein assay and protein samples were separated by electrophoresis on SDS-PAGE and transferred to a polyvinylidene difluoride membrane. The membranes were blocked with 5% milk and incubated overnight with the appropriate primary antibodies (anti-TXNIP, anti-NLRP3, and anti-caspase-1 antibody), respectively, followed by incubation with the corresponding secondary antibodies. The blots were visualized with ECL-plus reagent. GAPDH was used as the internal loading control.

### 2.12. Cell Viability and Lactate Dehydrogenase (LDH) Activity

Cell viability was determined by using a cell counting kit-8 (CCK-8) assay, according to the manufacturer's instructions. HK-2 cells (1 × 10^5^ cells/well) were plated into 96-well plates and pretreated with various conditions (NG, HG, NH/R, HH/R, HH/R-siRNA, HH/R-scrambled siRNA, and HH/R-RES) as described, following which, 10 *µ*L CCK-8 (Beyotime: C0037, China) was added and cells were incubated for 4 hours, and the absorbance was measured at 450 nm with an ELISA assay plate reader. LDH content was measured by LDH Cytotoxicity Assay Kit (Jiancheng Biotech, Nanjing, China).

### 2.13. Apoptosis Assay

After reoxygenation, cells were trypsinized, washed twice with PBS, and resuspended in binding buffer. The percentage of apoptosis was evaluated by using an Annexin V-APC/7-AAD detection kit (Nanjing KeyGen Biotech, Nanjing, China) according to the manufacturer's instructions. Cells were stained with Annexin V-APC and 7-AAD for 15 min in the dark. Samples were assayed by flow cytometry with the FACScan system (BD Biosciences). Apoptotic cells were defined as the cells situated in the right two quadrants of each plot and the percentages were determined by flow cytometry.

### 2.14. Statistical Analysis

The data are reported as means ± SE. Statistical significance was assessed by one-way or two-way ANOVA followed by Tukey's test for multiple comparisons (GraphPad Prism 5.0). Two-way ANOVA was used for testing differences among the multiple experimental groups between the types of injury (sham versus I/R) across the groups of rats (diabetes versus nondiabetes, for the animal experiments). *P* < 0.05 was considered to be statistically significant.

## 3. Results

### 3.1. General Characteristics of the Experimental Animals before I/R Modeling

As shown in Table 1, STZ-induced diabetic rats had obvious diabetic symptoms of hyperglycemia, polydipsia, polyphagia, and weight loss. The plasma glucose of the diabetic rats increased but their body weight decreased compared to nondiabetic rats. RES treatment had no significant effect on body weight compared to DS group, but plasma glucose was significantly elevated in the RES group compared to NS group but lower than DS group (Table 1).

### 3.2. STZ-Induced Diabetic Rats Exhibit Aggravated Ischemia AKI-Induced Kidney Dysfunction

**Figure 1:**
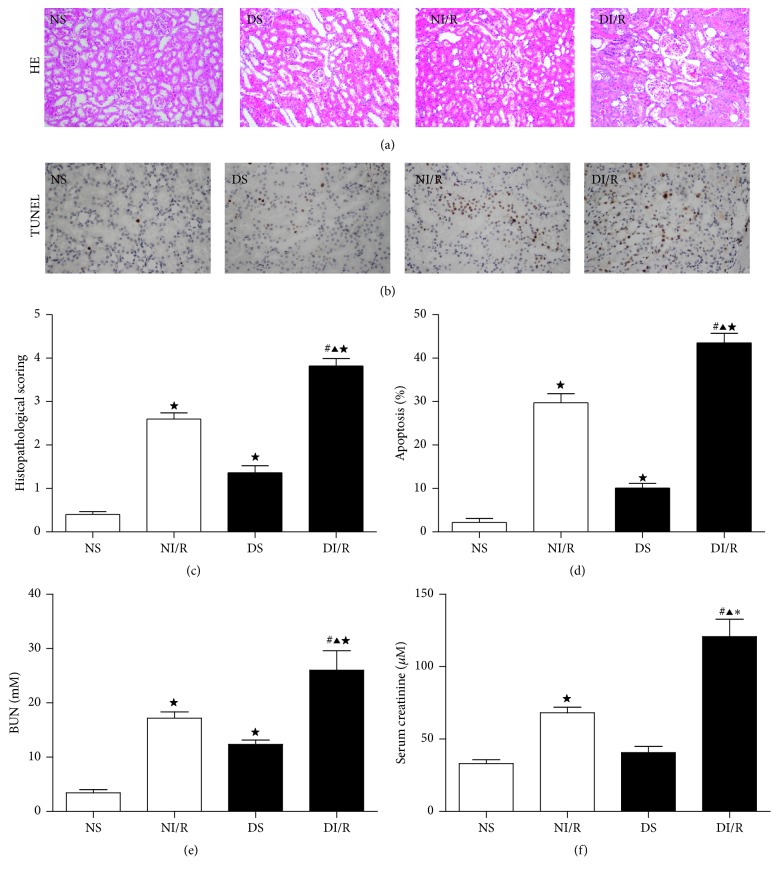
Induction of diabetes in male SD rats with STZ. The rats were subjected to sham operation or renal ischemia/reperfusion injury (I/R48). Renal tissues for hematoxylin and eosin staining and semiquantification of tubular damage (a, c). TUNEL assay apoptosis% (b, d). Blood samples were collected to measure BUN (e) and serum creatinine (f). The data in (c, d, e, f) are means ± SE (*n* = 6). ^★^ *P* < 0.05 versus NS group; ^#^ *P* < 0.05 versus DS group; ^▲^ *P* < 0.05 versus NI/R group. NS and DS: nondiabetic and STZ-induced diabetic rats were subjected to sham operation. NI/R and DI/R: nondiabetic and STZ-induced diabetic rats were subjected to 25 min ischemia followed by 48 h reperfusion. DI/R-RES: STZ-induced diabetic rats that underwent I/R were treated with RES (10 mg/kg, ip daily) for 7 consecutive days before renal ischemia-reperfusion.

As shown in Figure 1, we initially compared sensitivity of I/R injury to STZ-induced diabetic rat (DM) and nondiabetic rat without STZ (ND). For functional analysis, renal histology revealed significantly more tissue damage in DI/R group. I/R48 induced severe tubular dilation and interstitial edema, tubular epithelial swelling, loss of brush border, vacuolar degeneration, necrotic tubules, cast formation, and desquamation in DM rats, while, in ND rats, there were fewer injured tubules and the injury in each tubule was less severe as compared to DI/R rats (Figure 1(a)). Quantitatively, tubular damage was significantly higher at I/R48 in DI/R group than NI/R group (by 1.5-fold) (Figure 1(c)). Moreover, apoptotic cells assays by TUNEL were rare in kidney tissues of both NS and DS groups, and after I/R48, the percentage of apoptotic cells was significantly higher in DI/R group than NI/R group (by 1.5-fold) (Figures 1(b) and 1(d)). Compared to NS and DS groups, I/R48 resulted in marked increase with BUN (Figure 1(e)) and serum creatinine (Figure 1(f)) in both NI/R and DI/R groups, but the BUN and serum creatinine were significantly higher in DI/R group than NI/R group.

### 3.3. STZ-Induced Diabetic Rats Exhibit Increased Oxidative Stress in Kidney after Ischemia AKI

**Figure 2:**
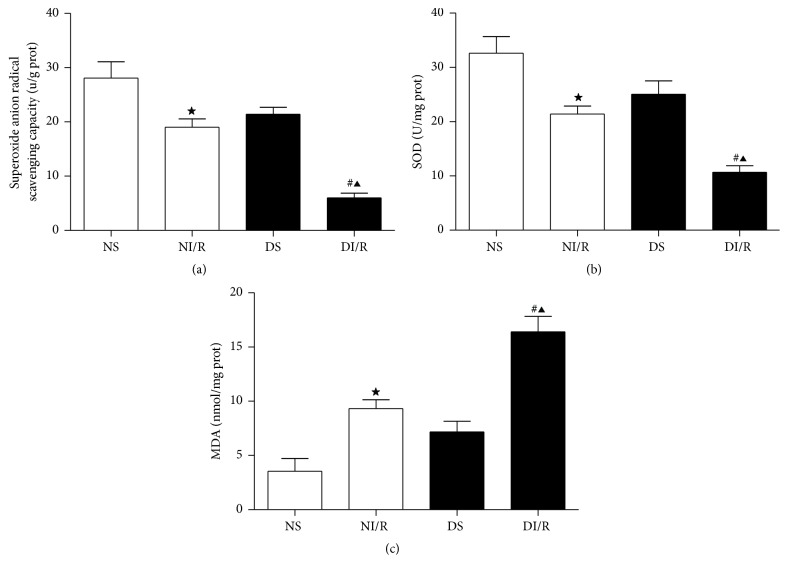
STZ-induced diabetes enhances oxidative stress in rats subjected to ischemia AKI. Kidney tissues were collected to measure superoxide anion radical scavenging capacity (a), kidney superoxide dismutase (SOD) contents (b), and malondialdehyde (MDA) activity (c). The data in (a, b, c) are means ± SE (*n* = 6). ^★^ *P* < 0.05 versus NS group; ^#^ *P* < 0.05 versus DS group; ^▲^ *P* < 0.05 versus NI/R group. NS and DS: nondiabetic and STZ-induced diabetic rats were subjected to sham operation. NI/R and DI/R: nondiabetic and STZ-induced diabetic rats were subjected to 25 min ischemia followed by 48 h reperfusion. DI/R-RES: STZ-induced diabetic rats that underwent I/R were treated with RES (10 mg/kg, ip daily) for 7 consecutive days before renal ischemia-reperfusion.

As shown in Figure 2, we determined superoxide anion radical scavenging capacity which indicated the general scavenging ability of kidney tissue on superoxide anion free radicals in each group and found that I/R48 resulted in reducing superoxide anion radical scavenging capacity (Figure 2(a)) in both NI/R and DI/R groups, but the superoxide anion radical scavenging capacity was significantly lower by 68.4% in DI/R group than NI/R group. In addition, antioxidant enzymes SOD content, which is a natural superoxide free radical scavenging factor, was significantly decreased in both DI/R and NI/R groups as compared to each sham group. Furthermore, SOD was markedly lower by 50% in DI/R group than NI/R group (Figure 2(b)). In contrast, MDA activity was used as a biomarker to measure the level of oxidative stress and showed a significant increase of MDA production in both DI/R and NI/R groups as compared to each sham group, but MDA was markedly higher in DI/R group than NI/R group (by 1.76-fold) (Figure 2(c)).

### 3.4. Effects of RES on Renal Function and Oxidative Stress in the STZ-Induced Diabetic Kidneys

**Figure 3:**
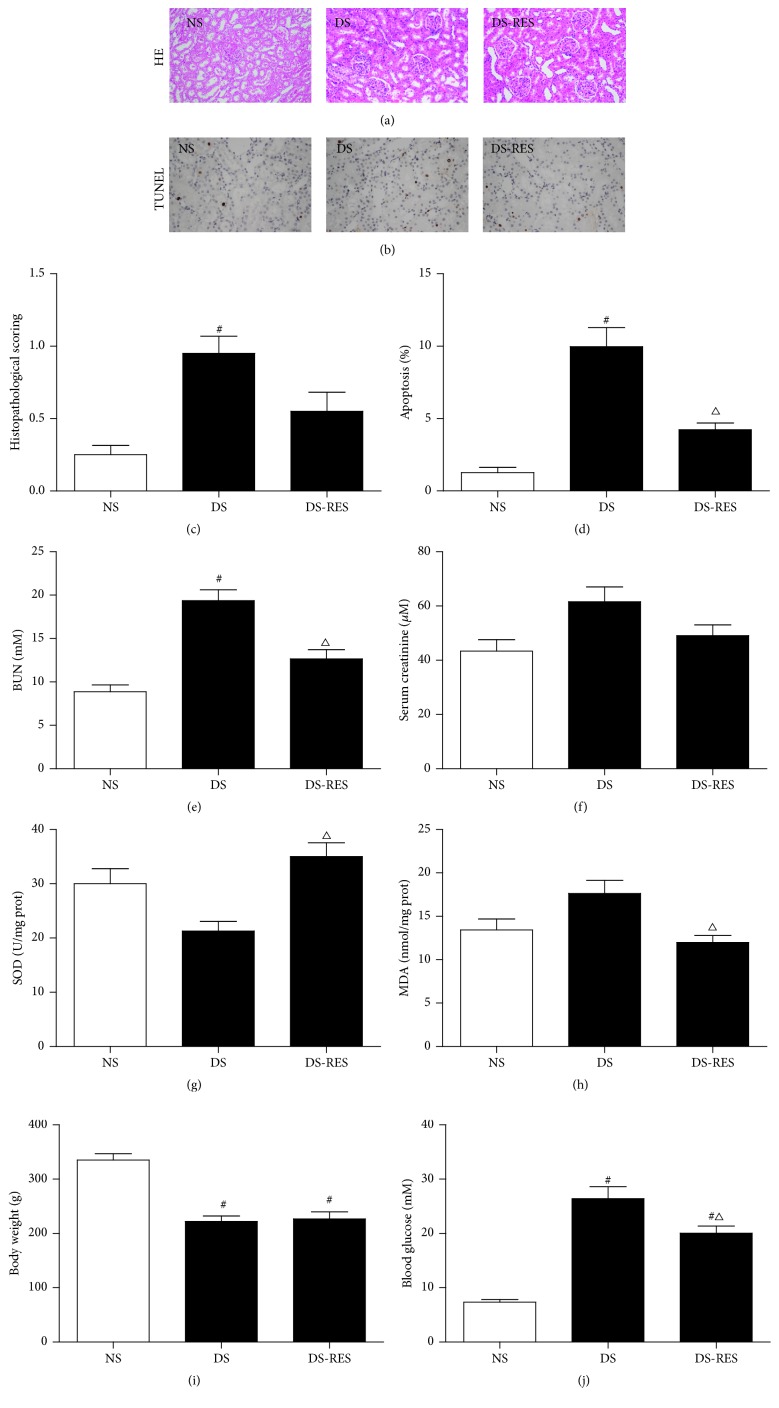
RES treatment of STZ-induced diabetes ameliorates renal dysfunction and oxidative stress level. After RES administration for 7 consecutive days (daily ip 10 mg/kg RES), kidney tissues were collected for H&E staining and the histological damage score (a, c) and TUNEL assay of apoptosis (b, d). Blood samples for measuring BUN (e), serum creatinine (f), kidney tissues for analysis of SOD content (g) and MDA production (h), body weight (i), and plasma glucose (j) were examined. The data in (c–j) are means ± SE (*n* = 4). ^#^ *P* < 0.05 versus NS group; ^△^ *P* < 0.05 versus DS group. NS and DS: nondiabetic and STZ-induced diabetic rats were subjected to sham operation. NI/R and DI/R: nondiabetic and STZ-induced diabetic rats were subjected to 25 min ischemia followed by 48 h reperfusion. DS-RES: STZ-induced diabetic rats that underwent sham operation were treated with RES (10 mg/kg, ip daily) for 7 consecutive days.

As shown in Figure 3, histology score (Figures 3(a) and 3(c)), apoptosis (Figures 3(b) and 3(d)), and BUN (Figure 3(e)) levels were significantly increased in the sham group of diabetic rats (DS). Meanwhile, the indicators of oxidative stress including the activities of SOD (Figure 3(g)) and MDA (Figure 3(h)) were slightly but not significantly increased in the kidney tissues of DS rats as compared with NS rats (*P* > 0.05). RES administration markedly ameliorated apoptosis (Figures 3(b) and 3(d)) and BUN (Figure 3(e)) levels. There was no remarkable attenuation on the histology score (Figure 3(c)) and serum creatinine (Figure 3(f)) levels after RES treatment in the STZ-induced DM rats. Furthermore, treatment with the RES significantly increased the SOD levels (Figure 3(g)) and decreased the MDA levels (Figure 3(h)) in the diabetic rats. Additionally, the plasma glucose of the diabetic rats increased but their body weight decreased as compared to nondiabetic rats. RES treatment had no significant effects on body weight but significantly attenuated plasma glucose as compared to DS group, though the blood glucose in RES group was still significantly higher than DS group (Figures 3(i) and 3(j)). Of note, RES had no significant effects on blood glucose, body weight, BUN, serum creatinine, SOD, and MDA in the normal rats (data not shown).

### 3.5. RES Treatment of STZ-Induced Diabetic Rats Attenuates the Ischemia AKI Sensitivity and Oxidative Stress Level

**Figure 4:**
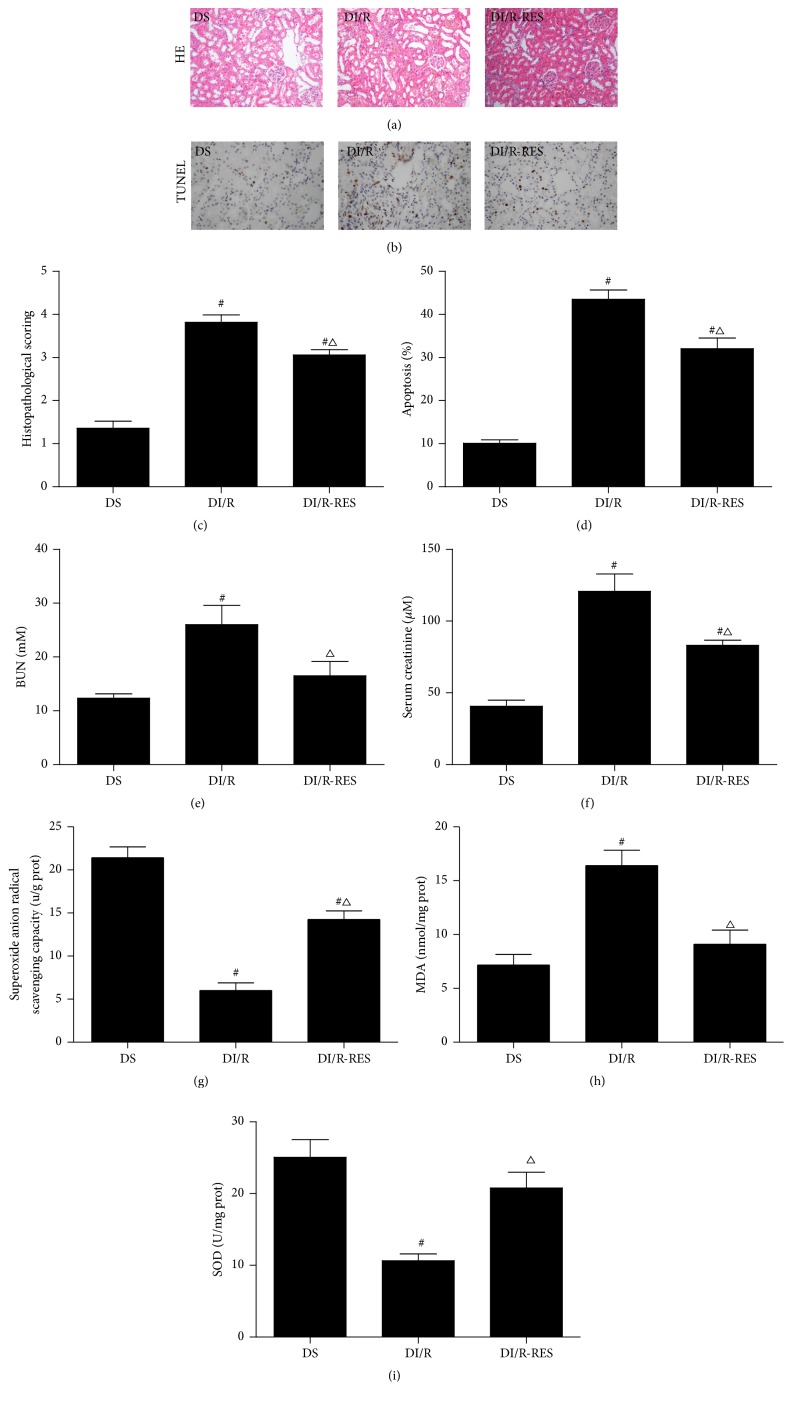
RES treatment of STZ-induced diabetes reduces AKI sensitivity and oxidative stress level. After I/R 48 hours, kidney tissues were collected for H&E staining and the histological damage score (a, c) and TUNEL assay of apoptosis (b, d) were examined; blood samples were used to measure BUN (e), serum creatinine (f) levels, and kidney tissues for analysis of superoxide anion radical scavenging capacity (g), MDA production (h), and SOD content (i). The data in (c–i) are means ± SE (*n* = 6). ^#^ *P* < 0.05 versus DS group; ^△^ *P* < 0.05 versus DI/R group. NS and DS: nondiabetic and STZ-induced diabetic rats were subjected to sham operation. NI/R and DI/R: nondiabetic and STZ-induced diabetic rats were subjected to 25 min ischemia followed by 48 h reperfusion. DI/R-RES: STZ-induced diabetic rats that underwent I/R were treated with RES (10 mg/kg, ip daily) for 7 consecutive days before renal ischemia-reperfusion.

As shown in Figure 4, RES significantly attenuated the AKI sensitivity of STZ-induced diabetic rats as indicated by histology and apoptosis, BUN (*P* > 0.05 versus DS group), and serum creatinine (Figures 4(a)–4(f)).

We also examined the effect of RES on oxidative stress level after I/R 48 hours and found that there was a significant increase of superoxide anion radical scavenging capacity (Figure 4(g)) and SOD content (Figure 4(i)) in RES-treated group as compared to DI/R group. The MDA production in RES-treated group was significantly decreased as compared to DI/R group (Figure 4(h)).

### 3.6. STZ-Induced Diabetes Stimulates TXNIP Expression and NLRP3 Inflammasome Activation following Renal I/R 48 Hours and Subsequent Effect of RES on TXNIP Expression and Inflammasome Activation

**Figure 5:**
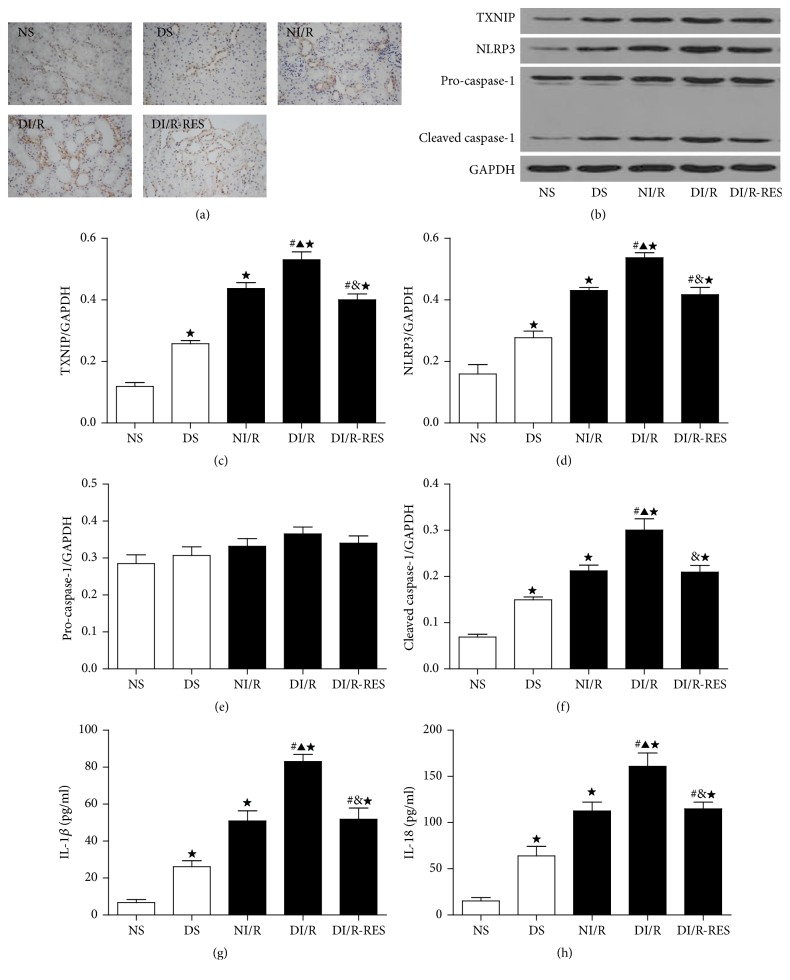
STZ-induced diabetes induces TXNIP expression and NLRP3 inflammasome activation following I/R 48. TXNIP expression was examined by IHC (a), representative blots (b), and quantitative analysis of Western blots for TXNIP (c), NLRP3 (d) and pro-caspase-1 and cleaved caspase-1 (e-f), and release of IL-1*β* and IL-18 by ELISA (g-h). The data in (c–h) are means ± SE (*n* = 5). ^★^ *P* < 0.05 versus NS group; ^#^ *P* < 0.05 versus DS group; ^▲^ *P* < 0.05 versus NI/R group; ^&^ *P* < 0.05 versus DI/R group. NS and DS: nondiabetic and STZ-induced diabetic rats were subjected to sham operation. NI/R and DI/R: nondiabetic and STZ-induced diabetic rats were subjected to 25 min ischemia followed by 48 h reperfusion. DI/R-RES: STZ-induced diabetic rats that underwent I/R were treated with RES (10 mg/kg, ip daily) for 7 consecutive days before renal ischemia-reperfusion.

As TXNIP is known to be involved in oxidative stress and diabetic complications, we determined kidney TXNIP expression by IHC (Figure 5(a)) and Western blot (Figures 5(b) and 5(c)) in ND rats and DM rats treated after I/R injury. In the sham groups, the TXNIP protein expression was significantly higher in DS than NS group. After I/R 48, both NI/R group and DI/R group stimulated kidney TXNIP expression as compared to each sham group. Furthermore, DI/R group showed a significant increase in TXNIP content compared with NI/R group (by 1.21-fold). The data suggested that ischemia AKI-induced TXNIP was enhanced by STZ-induced diabetes. Moreover, RES administration (DI/R-RES group) significantly decreased the expression of TXNIP by 24.6% compared to DI/R group. We also examined the effect of TXNIP on NLRP3 activation; after ischemia AKI injury, kidney NLRP3 inflammasome expression was dramatically increased in both DI/R rats and NI/R rats as compared to rats of each sham group. Furthermore, compared to NI/R group, the DI/R group showed significantly elevated levels of NLRP3 (by 1.25-fold) (Figure 5(d)). Meanwhile, increase in NLRP3 protein activation was accompanied by marked induction of cleavage of caspase-1 expression (Figure 5(e)) and IL-1*β* and IL-18 (Figures 5(f) and 5(g)) release. Moreover, RES treatment attenuated NLRP3 inflammasome protein activation at 48 hours after ischemia in DM rats while concomitantly reducing the expression of caspase-1 and release of IL-1*β* and IL-18 (Figures 4(b), 4(d), and 4(f)–4(h)). However, pro-caspase-1 (Figure 5(e)) had no significant change among all groups.

### 3.7. Inhibition of TXNIP Improves the Viability and Injury of HK-2 Cells Impaired by High Glucose (HG) and Hypoxia/Reoxygenation (HH/R)

**Figure 6:**
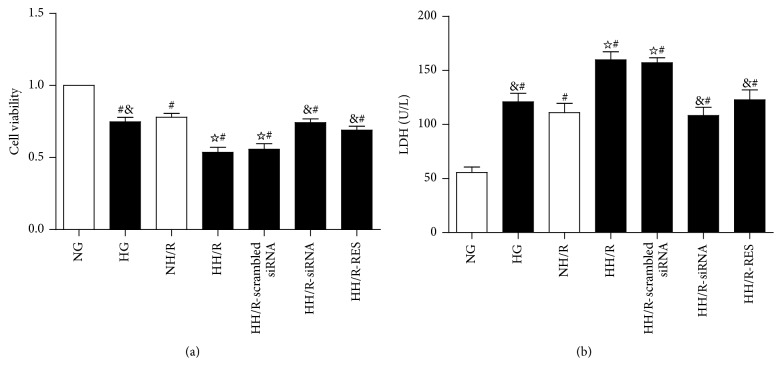
HK-2 cells injury after 4 hours of hypoxia followed by 2 hours of reoxygenation under high glucose stimulation. Effects of TXNIP blockage on cell viability assessed by CCK-8 (a); lactate dehydrogenase (LDH) release (b). The data in (a-b) are means ± SE (*n* = 6). ^#^ *P* < 0.05 versus NG group; ^☆^ *P* < 0.05 versus NH/R group; ^&^ *P* < 0.05 versus HH/R-scrambled siRNA group; NG: normal glucose (5.6 mM); HG: high glucose (30 mM). NH/R: hypoxia (4 h)/reoxygenation (2 h) under NG condition; HH/R: hypoxia (4 h)/reoxygenation (2 h) under HG condition. HH/R-RES: HH/R pretreated by RES (50 *μ*M) for 72 h with the high glucose incubation. HH/R-siRNA: TXNIP protein was inhibited by transfection with TXNIP siRNA before HH/R; HH/R-scrambled siRNA: scrambled siRNA used as control before HH/R.

Following 4 hours of hypoxia and 2 hours of reoxygenation, cell viability was significantlyreduced (Figure 6(a)) and cellular LDH activity was increased (Figure 6(b)) in HK-2 cells exposed to high glucose. Both transfection of HK-2 cells with TXNIP siRNA and RES treatment markedly alleviated HH/R-induced reduction of cell viability and elevation of cellular LDH activity.

### 3.8. Changes of TXNIP/NLRP3 Signaling in HK-2 Cells Exposed to High Glucose (HG) and Hypoxia/Reoxygenation (HR)

**Figure 7:**
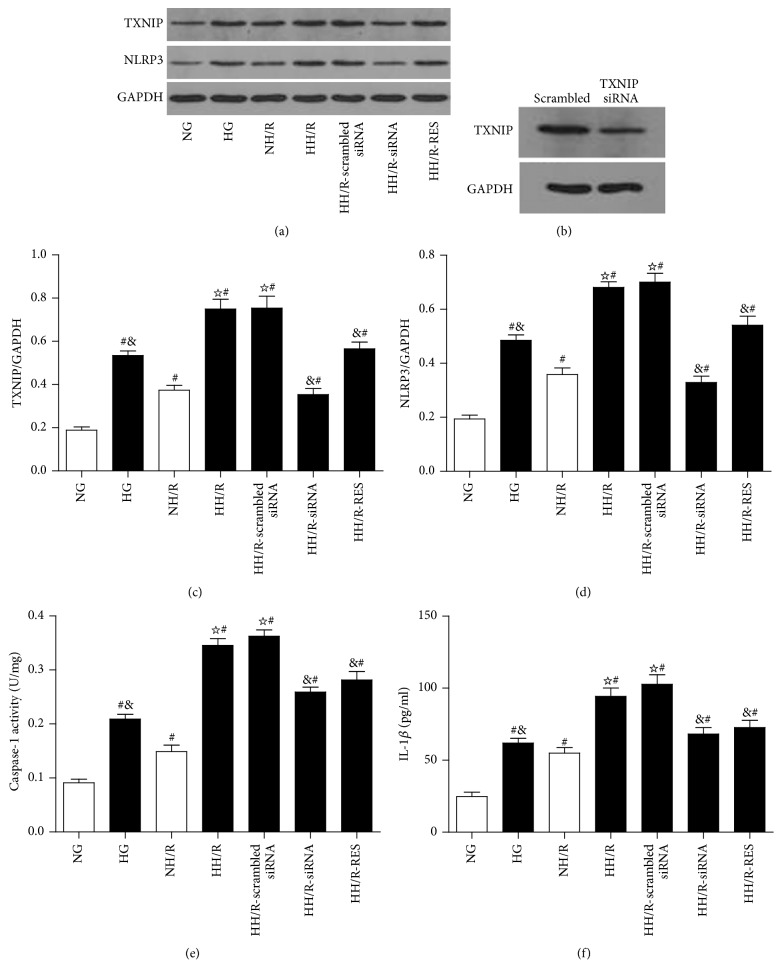
Western blot analysis of TXNIP and NLRP3 protein expression in cultured HK-2 cells treated by normal glucose (5.5 mM), high glucose (30 mM), and NG + mannitol, respectively, for 72 hours, then following 4 hours of hypoxia and 2 hours of reoxygenation in HK-2 cells under high glucose stimulation with or without TXNIP siRNA and RES treatment, respectively. Representative blots (a) and quantitative analysis of Western blots for TXNIP (c) and NLRP3 (d), activity of caspase-1 (e), level of IL-1*β* (f), and Western blot of TXNIP gene knockdown in HK-2 cells (b). The data in (c–f) are means ± SE (*n* = 5). ^#^ *P* < 0.05 versus NG group; ^☆^ *P* < 0.05 versus NH/R group; ^&^ *P* < 0.05 versus HH/R-scrambled siRNA group.

**Figure 8:**
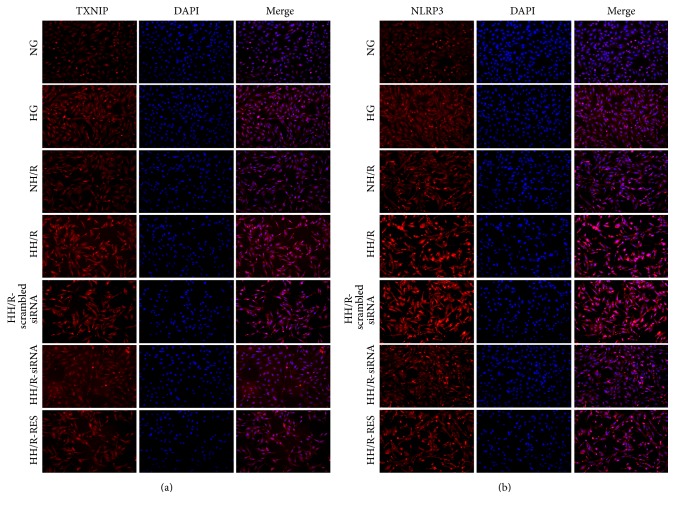
Immunofluorescence staining of TXNIP (a) and NLRP3 (b). NG: normal glucose (5.6 mM); HG: high glucose (30 mM). NH/R: hypoxia (4 h)/reoxygenation (2 h) under NG conditions; HH/R: hypoxia (4 h)/reoxygenation (2 h) under HG conditions. HH/R-RES: HH/R pretreated by RES (50 *μ*M) for 72 h with the high glucose incubation. HH/R-siRNA: TXNIP protein was inhibited by transfection with TXNIP siRNA before HH/R; HH/R-scrambled siRNA: scrambled siRNA used as control before HH/R.

At first, we chose NG plus mannitol (24.4 mM) as an osmotic control, and there was no significant difference between the NG group and NG plus mannitol group (data not shown). However the expressions of TXNIP protein (Figures 7(a) and 7(c)) and NLRP3 protein (Figures 7(a) and 7(d)) as well as the activity of caspase-1 (Figure 7(e)) and IL-1*β* level (Figure 7(f)) were upregulated in response to high glucose (30 mM) stimulation for 72 hours. Following 4 hours of hypoxia and 2 hours of reoxygenation, TXNIP protein expression was significantly higher in HK-2 cells exposed to high glucose than normal glucose (by 2-fold) (Figures 7(a) and 7(c)), while NLRP3 (Figures 7(a) and 7(d)) expression and the activity of caspase-1 (Figure 7(e)) and IL-1*β* level (Figure 7(f)) had similar changes as TXNIP. Transfection of HK-2 cells with specific siRNA decreased TXNIP protein expression by more than 70% (Figure 7(b)). In line with the* in vitro* experiments, following transfection of TXNIP siRNA, TXNIP (Figures 7(a) and 7(c)) and NLRP3 (Figures 7(a) and 7(d)) protein expressions were decreased by 53.1%, and the activity of caspase-1 (Figure 7(e)) and the level of IL-1*β* were also reduced (Figure 7(f)) in HK-2 cells after exposure to high glucose following H/R. Meanwhile, RES treatment significantly decreased TXNIP protein expression by 35.2% (Figures 7(a) and 7(c)) and correspondingly decreased NLRP3 protein expression by 32.8% (Figures 7(a) and 7(d)) and the activity of caspase-1 (Figure 7(e)) and level of IL-1*β* (Figure 7(f)) as compared to HH/R group. Subsequently, the expressions of TXNIP (Figure 8(a)) and NLRP3 (Figure 8(b)) were determined by immunofluorescence staining. Obvious increases of TXNIP and NLRP3 expression were induced by both HG and H/R. Moreover, HH/R enhanced the above effects as compared to NH/R group, whereas transfection of TXNIP siRNA and treatment with RES inhibited the expression of TXNIP and NLRP3 under HH/R.

### 3.9. The Oxidative Stress Status and Apoptosis in HK-2 Cells Exposed to High Glucose (HG) and H/R

**Figure 9:**
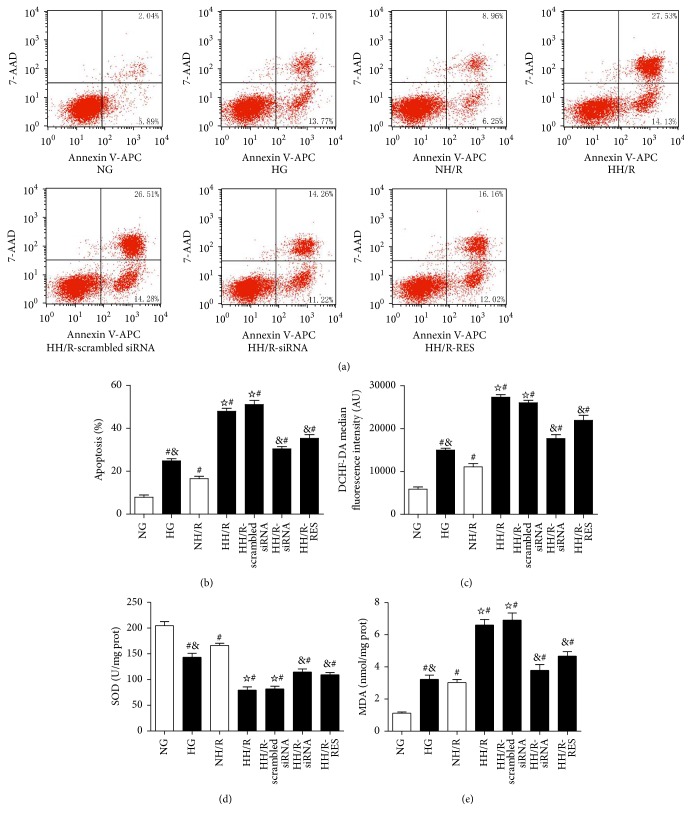
Effect of TXNIP inhibition on the HH/R-induced apoptosis and oxidative stress in HK-2 cells. Apoptotic cells were defined as the cells in the right two quadrants of each plot and the percentages were determined by flow cytometry (a, b); intracellular ROS was detected by flow cytometry (c); SOD (d) and MDA (e) were detected by microplate reader. The data in (b–e) are means ± SE (*n* = 5). ^#^ *P* < 0.05 versus NG group; ^☆^ *P* < 0.05 versus NH/R group; ^&^ *P* < 0.05 versus HH/R-scrambled siRNA group. NG: normal glucose (5.6 mM); HG: high glucose (30 mM). NH/R: hypoxia (4 h)/reoxygenation (2 h) under NG condition; HH/R: hypoxia (4 h)/reoxygenation (2 h) under HG condition. HH/R-RES: HH/R pretreated by RES (50 *μ*M) for 72 h with the high glucose incubation. HH/R-siRNA: TXNIP protein was inhibited by transfection with TXNIP siRNA before HH/R; HH/R-scrambled siRNA: scrambled siRNA used as control before HH/R.

To study the mechanism of the AKI sensitivity of HG and H/R, the HK-2 cells were cultured for 72 hours in 30 mM glucose (HG). These cells were then subjected to 4 hours of hypoxia and 2 hours of reoxygenation. Our studies demonstrated that both HG and H/R could induce higher apoptosis levels (Figures 9(a) and 9(b)) and oxidative stress (Figures 9(c)–9(e)); moreover, HH/R induced significantly increased apoptosis levels (Figures 9(a) and 9(b)) and oxidative stress (Figures 9(c)–9(e)) as compared to NH/R. Both knockdown of TXNIP and pretreatment with RES significantly inhibited ROS generation (by 32.1% and 15.7%, resp.) (Figure 9(c)) in HK-2 cells that were exposed to HG combined H/R, while the SOD content (Figure 9(d)) significantly increased (by 1.4-fold and 1.34-fold, resp.) and MDA (Figure 9(e)) significantly decreased (by 45.2% and 32.5%, resp.) as compared to HH/R group. Similarly, after HH/R, both TXNIP siRNA transfection and pretreatment with RES significantly decreased the percentage of apoptotic cells (by 40.5% and 30.9%, resp.) (Figures 9(a) and 9(b)) compared to HH/R group.

## 4. Discussion

Using STZ-induced diabetic rat models as well as high glucose cultured HK-2 cells, our study provides evidence for increased ischemia AKI sensitivity in DM. Mechanistically, the injury sensitivity involves upregulation expression of TXNIP protein which resulted in activation of NLRP3 inflammasome. Both* in vitro* and* in vivo* decrease of TXNIP expression by RES treatment or RNA interference blocked TXNIP expression and subsequently inhibited NLRP3 inflammasome activation in response to ischemia-induced AKI in DM. These findings demonstrate the critical role of TXNIP which subsequently triggered activation of NLRP3 inflammasome in AKI sensitivity in DM.

Diabetic patients with AKI have an increased risk of advanced CKD, and AKI is an independent risk factor of kidney disease progression [7]. Renal I/R is one of the major causes of AKI, while cardiovascular surgery and renal transplantation may also cause ischemia AKI in clinical settings [39]. In animal models, the exaggerated vulnerability of the diabetic kidney to the ischemic insult could be attributed to activation of proinflammatory cytokine pathways [40]. Previous research revealed that this susceptibility was attributed to the oxidative and nitrosative stress [41]. Furthermore, a recent study demonstrated the role of apoptosis in the susceptibility of diabetic models to AKI [42]. Consistent with a previous study [43], herein, after 4 weeks of STZ-induced diabetic rats, the diabetic rats showed higher levels of BUN, tubule injury score, and apoptosis than nondiabetic rats, indicating that diabetic kidney was already significantly injured before I/R. However, the diabetic rats were also more sensitive to renal I/R injury as compared with nondiabetic rats. Following I/R injury, renal dysfunction and apoptosis level were more severe in diabetic rats than in nondiabetic rats. Our current results suggested that at the cellular level the injury sensitivity may be due to cell viability and the release of LDH. TXNIP gene knockdown by siRNA or RES treatment could inhibit TXNIP and NLRP3 expression and reduce the apoptosis level and decrease the cellular LDH activity. We have demonstrated that diabetic or high glucose further promoted I/R- (H/R-) induced MDA content expression. In addition, both kidney and cell SOD contents were significantly impaired. Meanwhile, the superoxide anion radical scavenging capacity in diabetic kidney was declined. Specifically, in response to hypoxia and reoxygenation, high glucose-conditioned HK-2 cells showed significantly higher expression of TXNIP and NLRP3 inflammasome. Moreover, renal I/R stimulated TXNIP expression which induced higher NLRP3 activation and IL-1*β* (IL-18) release from diabetic kidneys than nondiabetic tissues. Importantly, we further showed that RES could decrease TXNIP expression and NLRP3 activation both* in vivo* and* in vitro*. To the best of our knowledge, this is the first report of the renal I/R injury sensitization through TXNIP/NLRP3 pathway by high glucose* in vitro* and diabetic* in vivo*.

The activation of NLRP3 inflammasomes has been implicated in various pathological conditions, ranging from metabolic syndrome and kidney diseases [44, 45]. Formation of NLRP3 inflammasome can increase the expression of cleaved caspase-1 and IL-1*β* (IL-18) and mature IL-1*β* that participate in the pathological process of renal I/R [15, 16], in addition to revealing its role in apoptosis [46]. Despite studies that have demonstrated that NLRP3 contributes to renal ischemia reperfusion (I/R) injury by a direct effect on renal tubular epithelium [17, 18], it remains unclear how NLRP3 is able to sense redox changes, particularly in renal tubular epithelium during ischemia AKI in diabetes. In our study, we reconfirmed that the NLRP3/cleaved caspase-1/IL-1*β*, IL-18 signal pathway was upregulated in the nondiabetic kidney following I/R and normal-glucose-cultured HK-2 cells following H/R. Moreover, there was significantly increased expression of NLRP3 inflammasome in diabetic kidney and high-glucose-cultured HK-2 cells after I/R and H/R, respectively, accompanied by increased tubular cells apoptosis. These results provide evidence that NLRP3 inflammasome plays a key role in the pathogenesis of ischemia AKI of diabetic models.

To date, some mechanisms concerning the NLRP3 inflammasome activation have been identified, for example, intracellular ROS, which are commonly produced in response to many NLRP3 activators [19], and TXNIP (also known as vitamin D_3 _upregulated protein-1 [VDUP-1] and thioredoxin-binding protein-2 [TBP-2]), the endogenous inhibitor and regulator of TRX [24]. The TRX-TXNIP system, as a major ROS-scavenging system, maintains intracellular redox balance [27]. Overexpression of TXNIP inhibits the activity of TRX and thereby can modulate the cellular redox state and promote oxidative stress [25, 26]. Liu et al. demonstrated that TXNIP-mediated NLRP3 inflammasome activation was involved in myocardial I/R injury [47]. Hyperglycaemia has been identified as an inducer of TXNIP expression in various cells including HK-2 [21, 22]; meanwhile, enhanced myocardial TXNIP expression that contributes to hyperglycaemia-aggravated oxidative stress and exacerbates cardiac injury following I/R has been reported [48]. TXNIP expression is markedly upregulated in human diabetes and diabetic complications [49, 50], indicating that TXNIP is a potential therapeutic target of diabetes. Recently Zhou et al. confirmed an important role for TXNIP in the pathogenesis of type-2 diabetes and showed that TXNIP binding to NLRP3 was essential for ROS-mediated inflammasome activation [23]. Recent work demonstrated that TXNIP induced NLRP3 expression, activation of caspase-1, and release of IL-1*β* in a model of hyperhomocysteinemia or hyperglycemia with podocyte injury [51, 52]. In our current study, to our knowledge, this is the first report that shows TXNIP expression was induced in kidney tissue or HK-2 cells under ischemia (hypoxia) and in diabetic (high glucose) combined with ischemia (hypoxia) conditions. Additionally, we sought to determine the effect of TXNIP and elucidated its relationship to NLRP3 inflammasome activation in the susceptibility to ischemic AKI in diabetes.

Several studies have demonstrated that RES exerts protective effects against I/R injury in the kidneys [31], as well as the liver and brain injury by reducing oxidative stress and due to inhibition of TXNIP expression [32, 33]. In the present study, we found that TXNIP protein expression was significantly reduced after siRNA transfection in cultured HK-2 cells. We also found that RES could decrease TXNIP expression both* in vitro* and* in vivo*. Both RES at a dose of 50 *µ*M and TXNIP siRNA markedly attenuated reduction of cell viability and increase of cellular LDH activity in high glucose and H/R treated HK-2 cells. Meanwhile, TRX-TXNIP interaction is an important antioxidant system and TXNIP is the endogenous inhibitor of cellular TRX [24, 27]. Previous researches showed that knockdown of TXNIP prevented high glucose (30 mM)-induced intracellular ROS generation and reversed the high glucose-induced suppression of TRX activity in HK-2 cells [22]. Further, knockdown of TXNIP in mouse mesangial cells also suppressed high glucose-induced apoptosis by the reduction of ROS [53]. Furthermore, gene silencing of TXNIP reduced hyperglycaemia-elevated ROS production and apoptosis in cardiomyocytes within H/R [48]. In our* in vitro* study, both TXNIP siRNA and RES treatment significantly prevented HH/R-induced excessive oxidative stress (indicated by ROS, MDA, and SOD) and apoptosis level in HK-2 cells, subsequently prevented HH/R-induced NLRP3 inflammasome formation, and hindered caspase-1 activation in HK-2 cells. Additionally, RES treatment ameliorated hyperglycemia-mediated renal dysfunction or diabetic nephropathy [30, 36, 54]. In our* in vivo* experiments, we found that RES treatment downregulated the blood glucose level in diabetic rats, which was consistent with previous studies [36, 54]; this insulin-like property protective effect required further exploration. Meanwhile, a recent study showed that TXNIP expression was increased in the glomerular lysate of DN mice [52]. Our study found that the expression of TXNIP was increased in the diabetic kidney about 4 weeks after induction with STZ. Moreover, RES treatment partly normalized renal dysfunction in diabetic rats in addition to attenuating oxidative stress which was indicated by histology score (*P* > 0.05), BUN, apoptosis%, MDA, and SOD activities. Next, STZ-induced diabetic rats were used to establish I/R injury models, and RES further ameliorated renal function and the ability of antioxidative stress, decreased TXNIP-mediated NLRP3 inflammasome activation and expression of cleaved caspase-1 protein, and impeded IL-1*β* maturation in I/R-induced AKI in diabetic models. Our results indicated that TXNIP-mediated NLRP3 inflammasome activation is a ROS-dependent way.

## 5. Conclusion

Our results demonstrate that TXNIP inhibition protects the diabetic kidney from I/R injury and diminishes the AKI sensitivity of diabetic kidney tissues by inhibiting oxidative stress and NLRP3 inflammasome activation. Our findings further suggest that TXNIP, an endogenous redox regulator, may represent an important future target to develop newer therapeutics in diabetic patients that can reduce AKI sensitivity of kidney tissues.

## Figures and Tables

**Table 1:** General characteristics of the experimental animals before ischemia/reperfusion modeling.

	NS (*n* = 6)	NI/R (*n* = 7)	DS (*n* = 7)	DI/R (*n* = 7)	DI/R-RES (*n* = 8)
Blood glucose (mM)	8.15 ± 0.58	7.44 ± 0.6	27.17 ± 1.1^★^	25.24 ± 1.5^★^	18.9 ± 0.86^★#^
Body weight (g)	359.67 ± 9.55	366.43 ± 9.81	217.43 ± 9.92^★^	204.86 ± 12.33^★^	224 ± 9.89^★^

The data in the table are means ± SE (*n* = 6–8), ^★^ *P* < 0.05 versus NS group, and ^#^ *P* < 0.05 versus DS group.

NS and DS: nondiabetic and STZ-induced diabetic rats were subjected to sham operation. NI/R and DI/R: nondiabetic and STZ-induced diabetic rats were subjected to 25 min ischemia followed by 48 h reperfusion. DI/R-RES: STZ-induced diabetic rats that underwent I/R were treated with RES (10 mg/kg, ip daily) for 7 consecutive days before renal ischemia-reperfusion.
